# A Protein Extract from Chicken Reduces Plasma Homocysteine in Rats

**DOI:** 10.3390/nu7064498

**Published:** 2015-06-04

**Authors:** Vegard Lysne, Bodil Bjørndal, Rita Vik, Jan Erik Nordrehaug, Jon Skorve, Ottar Nygård, Rolf K. Berge

**Affiliations:** 1 Department of Clinical Science, University of Bergen, 5020 Bergen, Norway; E-Mails: vegard.lysne@helse-bergen.no (V.L.); bodil.bjorndal@k2.uib.no (B.B); rita.vik@k2.uib.no (R.V.); jan.erik.nordrehaug@sus.no (J.E.N.); jon.skorve@k2.uib.no (J.S.); ottar.nygard@helse-bergen.no (O.N.); 2 Department of Heart Disease, Haukeland University Hospital, 5021 Bergen, Norway; 3 KG Jebsen Centre for Diabetes Research, University of Bergen, 5020 Bergen, Norway

**Keywords:** chicken protein, homocysteine, one-carbon metabolism, peroxisome proliferator activated receptor, Wistar rats

## Abstract

The present study aimed to evaluate effects of a water-soluble protein fraction of chicken (CP), with a low methionine/glycine ratio, on plasma homocysteine and metabolites related to homocysteine metabolism. Male Wistar rats were fed either a control diet with 20% w/w casein as the protein source, or an experimental diet where 6, 14 or 20% w/w of the casein was replaced with the same amount of CP for four weeks. Rats fed CP had reduced plasma total homocysteine level and markedly increased levels of the choline pathway metabolites betaine, dimethylglycine, sarcosine, glycine and serine, as well as the transsulfuration pathway metabolites cystathionine and cysteine. Hepatic mRNA level of enzymes involved in homocysteine remethylation, methionine synthase and betaine-homocysteine *S*-methyltransferase, were unchanged, whereas cystathionine gamma-lyase of the transsulfuration pathway was increased in the CP treated rats. Plasma concentrations of vitamin B2, folate, cobalamin, and the B-6 catabolite pyridoxic acid were increased in the 20% CP-treated rats. In conclusion, the CP diet was associated with lower plasma homocysteine concentration and higher levels of serine, choline oxidation and transsulfuration metabolites compared to a casein diet. The status of related B-vitamins was also affected by CP.

## 1. Introduction

Hypolipidemic effects of processed proteins or peptides have been reported in numerous different animal models [1,2,3,4,5,6,7]. These effects are most likely due to the amino acid composition or specific peptides in the different protein sources [1,8,9,10]. Recently, we have shown that a water-soluble protein extract of chicken (CP) had both hypotriglyceridemic and hypocholesterolemic effects in male Wistar rats [11]. The triglyceride lowering effect of the CP extract was associated with reduced hepatic triglyceride synthesis and increased mitochondrial oxidation of fatty acids, perhaps mediated by peroxisome proliferator-activated receptor (PPAR) α, of which the gene expression was increased. We also observed different plasma concentrations of several amino acids between the groups, with lower methionine and higher glycine and serine in animals fed CP.

**Figure 1:**
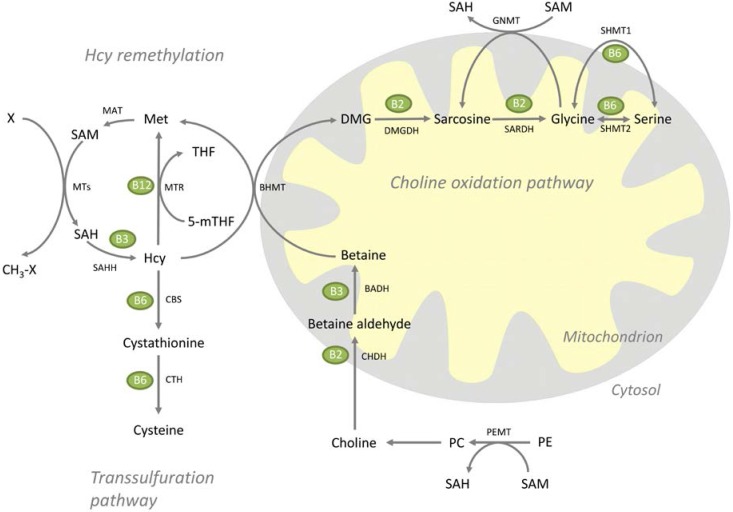
Overview of the homocysteine metabolism and the choline oxidation pathway. Homocysteine is catabolized through the transsulfuration pathway, or remethylated to methionine through either methionine synthase or betaine-homocysteine methyltransferase. The latter connects homocysteine metabolism to the choline oxidation pathway. BADH, betaine aldehyde dehydrogenase; BHMT, betaine homocysteine methyltransferase; CBS, cystathionine beta-synthase; CH_3_-X, methylated methyl acceptor; CHDH, choline dehydrogenase; CTH, cystathionine gamma lyase; CTP, cytidine triphosphate; DMG, dimethylglycine; DMGDH, dimethylglycine dehydrogenase; Hcy, homocysteine; Met, methionine; MTs, methyltransferases; MTR, methionine synthase; PC, phosphatidylcholine; PE, phosphatidylethanolamine; PEMT, phosphatidylethanolamine methyltransferase; SAH, S-adenosylhomocysteine; SAHH, *S*-adenosylhomocysteine hydrolase; SAM, S-adenosylmethionine; SARDH, sarcosine dehydrogenase; SHMT1, cytosolic serine hydroxymethyltransferase; SHMT2, mitochondrial serine hydroxymethyltransferase; THF, tetrahydrofolate; X, methyl acceptor.

Methionine serve as the precursor of the universal methyl donor S-adenosylmethionine (SAM), used in numerous methylation reactions throughout the body, with homocysteine (Hcy) being the end product [12]. The total amount of circulating homocysteine is referred to as total homocysteine (tHcy), and like the well-known lipid parameters, elevated circulating tHcy is also linked to increased risk of cardiovascular disease [13], though a causal relationship has been questioned due to the absence of clinical benefit after Hcy-lowering B-vitamin therapy [14]. Hcy has two metabolic fates. First, it can be permanently eliminated via the transsulfuration pathway to cystathionine and cysteine by the B6-dependent cystathionine beta-synthase (CBS) and cystathionine gamma lyase (CTH). Secondly, Hcy can be remethylated back to methionine either by methionine synthase (MS), which utilizes 5-methyltetrahydrofolate (mTHF) and cobalamin as cofactors, or by betaine-Hcy *S*-methyltransferase (BHMT), using betaine as the methyl donor [12]. BHMT-mediated remethylation of Hcy is linked to the choline oxidation pathway. In this reaction, betaine is converted to dimethylglycine (DMG), and further catabolized to sarcosine and glycine by the B2-dependent DMG dehydrogenase (DMGDH) and sarcosine dehydrogenase (SARDH), respectively [15,16]. Sarcosine can also be formed from glycine by the cytosolic enzyme glycine methyltransferase (GNMT), using SAM as methyl donor [17], and this is thought to modulate SAM levels by metabolizing excess SAM [18]. The interconversion between glycine and serine is provided by serine hydroxymethyltransferase (SHMT) [19]. The Hcy metabolism and the choline oxidation pathway are depicted in Figure 1.

The aim of the present study was to provide further insight into effects of a water-soluble protein extract of chicken with particular emphasis on diet related changes in Hcy metabolism. Thus, we measured the effects of the protein source on one-carbon metabolites and vitamin B status in plasma, as well as changes in hepatic gene expression. The results show substantial protein-induced alterations of plasma tHcy, as well as of B-vitamins, metabolites and gene expression related to homocysteine metabolism.

## 2. Materials and Methods

### 2.1. Animals and Diets

Male Wistar rats (Tactonic Europe A/S, Ry, Denmark) 12 weeks old, were housed in Makrolon type II cages, 3 animals per cage, in an open system. They were kept under standard laboratory conditions with temperature 22 ± 1 °C, dark/light cycles of 12/12 h, relative humidity 55% ± 5% and 20 air changes per hour. The animal study was conducted according to the Guidelines for the Care and Use of Experimental Animals, and the Norwegian State Board of Biological Experiments with Living Animals approved the protocol.

Animals were divided at random into 4 groups of 6 rats, and after 7 days of acclimatization, the control group were fed a 20% casein diet with 7% fat (5% lard and 2% soy oil) while in the intervention diets, casein was replaced with 6%, 14% or 20% CP (group 2–4), respectively. The composition of the diets, the procedures of making the water soluble CP extract and the amino acid profile of casein and CP are previously described [11], and the amino acid composition is given in Table 1. Notably, the 20% CP diet contains only half the amount of methionine, and has a 6-fold higher content of glycine compared to the casein diet, resulting in a 14-fold lower ratio of methionine to glycine. This resulted in a 57-fold lower ratio in plasma [11]. All groups had free access to tap water and feed during the 28 days experiment. Rat feed intake and weight gain were determined twice a week. Rats in the 20% CP group had a higher feed intake as compared to control, but gained less weight [11].

**Table 1:** Amino acid composition of the control diet and the 20% CP diet.

Amino Acid (mg/kg)	Control	20% CP
*Ala*	6.37	11.79
Arg	5.77	9.98
*Asx*	15.87	11.58
Glx	49.44	22.11
*Gly*	3.65	22.67
His	5.39	3.20
Hyp	0	11.86
*Ile*	11.20	4.11
*Leu*	19.65	8.49
*Lys*	17.15	7.69
*Met*	5.73	2.60
*Phe*	10.32	5.01
Pro	23.46	15.04
*Ser*	11.07	5.68
*Tau*	0.01	0.64
*Thr*	9.15	4.71
*Tyr*	8.24	2.34
*Val*	13.47	5.41

Ala, alanine; Arg, arginine; Asx, aspartic acid + aspargine; Glx, glutamic acid + glutamine; Gly, glycine; His, histidine; Hyp, hydroxyproline; Ile, isoleucine; Leu, leucine; Lys, lysine; Met, methionine; Phe, phenylalanine; Pro, proline; Ser, serine; Tau, taurine; Thr, threonine.

All rats were killed on day 28 after an overnight fast. They were anesthetized by inhalation of 2% Isofluorane (Forane, from Abbott Laboratories Ltd, Chicago, IL, USA) in an anesthesia chamber and thoracotomy, cardiac puncture, and exsanguination was performed. Plasma and liver samples were stored at −80 °C.

### 2.2. Biochemical Analyses

Methylmalonic acid (MMA), tHcy, sarcosine, serine and glycine were analyzed in plasma by gas chromatography- tandem mass spectrometry (GC-MS/MS) [20]. Plasma choline, betaine, DMG, methionine, cysteine, and creatinine, [21], and all vitamin B2, B3, and B6 forms (flavin mononucleotide (FMN), riboflavin, nicotinamide, *N*^1^-methylnicotinamide, pyridoxal phosphate (PLP), pyridoxal and pyridoxic acid) and cystathionine [22] were analyzed by liquid chromatography-tandem mass spectrometry (LC-MS/MS). Plasma folate and cobalamin were measured by microbiological assays [23,24]. All analyzes were performed at Bevital A/S (http://www.bevital.no; Bergen, Norway)

### 2.3. Gene Expression Analysis

Gene expression analyses were performed in the control group and the 20% CP group. Total cellular RNA was purified from 20 μg of frozen liver samples using the RNeasy Mini Kit (Qiagen, Hilden, Germany). The quantity of the RNA was measured spectrophotometrically using a NanoDrop 1000 (NanoDrop Products, Wilmington, DE, USA) and the quality of the RNA was analyzed using the Agilent 2100 Bioanalyzer (Agilent Technologies, Santa Clara, CA, USA). The Applied Biosystems High Capacity cDNA Reverse Transcription Kit with RNase inhibitor (Foster City, CA, USA) was used to obtain cDNA by reverse transcription of 1 µg RNA. Real-time PCR was performed in triplicates with Sarstedt 384 well multiply-PCR Plates (Sarstedt Inc., Newton, NC, USA) on the following genes, using probes and primers from Applied Biosystems: choline kinase alpha (Chka, Rn00567492_m1), choline kinase beta (Chkb, Rn01407345_m1), phosphatidylethanolamine *N*-methyltransferase (Pemt, Rn00564517_m1), choline dehydrogenase (Chdh, Rn01644299_m1), Bhmt (Rn00578255_m1), Dmgdh (Rn00594751), Sardh (Rn00454657_m1), Shmt1, soluble (Rn01751636_m1), Shmt2, mitochondria (Rn01768052_m1), Gnmt (Rn00567215_m1), Mtr (5-methyltetrahydrofolate-homocysteine methyltransferase, Rn00578368_m1), methyltetrahydrofolate-homocysteine methyltransferase reductase (Mtrr, Rn01409369m1), Cbs (Rn00560948_m1) and Cth (Rn00567128_m1).

Three different reference genes were included: 18s (Kit-FAM-TAMRA (Reference RT-CKFT-18s)) from Eurogentec, Belgium, glyceraldehyde-3-phosphate dehydrogenase (Gapdh, Taqman Rodent GAPDH, No. 4308313) and ribosomal protein, large, P0 (Rplp0, Rn00821065_g1) from Applied Biosystems. cDNA was generated from universal rat reference RNA (Agilent Technologies) and used in 5–6 serial dilutions as a standard curve in all gene runs. The NormFinder software was used to evaluate the reference genes [25], and data were normalized to 18 s. Finally, expression values relative to the control group were calculated by dividing all individual values with the control group mean, and the results are presented as relative values ± SDs.

### 2.4. Statistical Analysis

Data sets were analyzed using Prism Software (Graph-Pad Software, San Diego, CA, USA) to determine statistical significance. The results are shown as means of 6 animals per group with their standard deviations. For the metabolite analyses, between-group differences were tested by one way ANOVA, and Dunnet’s *post hoc* test was performed to evaluate statistical differences between the individual intervention groups toward the control group. For the gene expression analyses, normal distribution was determined by the Kolmogorov-Smirnov test (with Dallal-Wilkinson-Lilliefor *p* value), and *p*-values < 0.05 were considered not normally distributed. Unpaired *t*-test was performed to evaluate statistical differences between groups, or Mann Whitney test when values were not normally distributed. *p*-Values < 0.05 were considered significant.

## 3. Results

### 3.1. Metabolites

**Figure 2:**
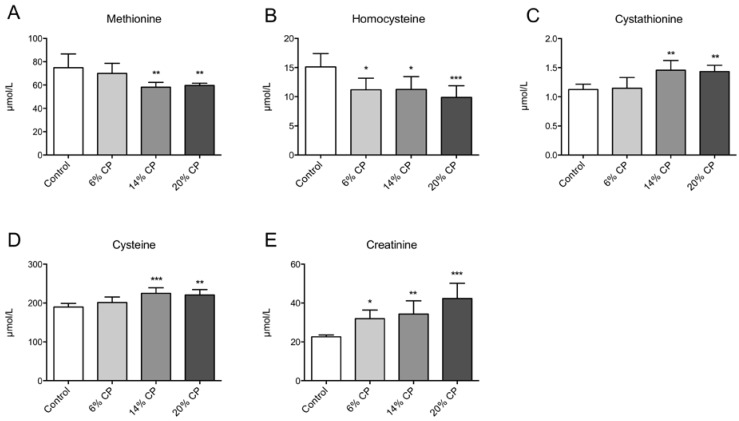
Plasma concentration of metabolites related to homocysteine metabolism. Rats were fed a 20% (w/w) casein control diet, or a diet containing 6%, 14% or 20% chicken protein (CP) for 4 weeks. Methionine (**A**), homocysteine (**B**), cysteine (**C**), cystathionine (**D**), and creatinine (**E**), were measured in fasting plasma samples. Values shown are means ± standard deviation (*n* = 6). One-way ANOVA with Dunnet’s *post hoc* test was used to determine values significantly different from control (* *p* < 0.05, ** *p* < 0.01, *** *p* < 0.001).

The plasma levels of methionine (Figure 2A) was reduced, and tHcy (Figure 2B) was lowered by CP. Metabolites of the transsulfuration pathway were also affected by CP treatment as the plasma concentrations of both cystathionine (Figure 2C) and cysteine (Figure 2D) were increased by 14% and 20% CP. In addition, CP increased the creatinine level in a dose-dependent manner (Figure 2E).

**Figure 3:**
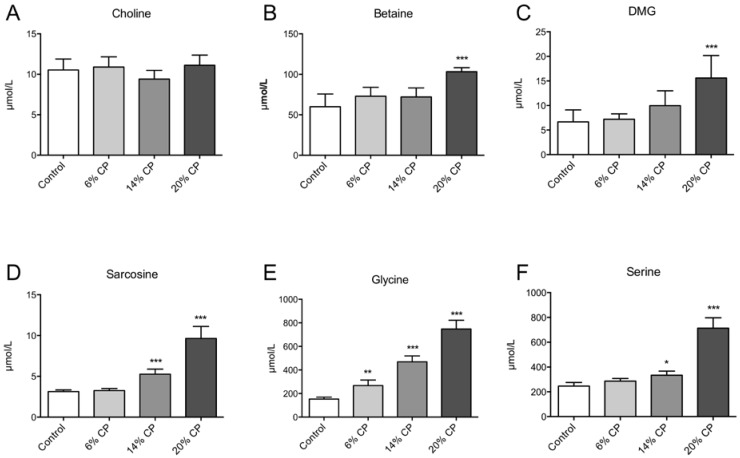
Plasma concentration of metabolites of the choline oxidation pathway. Rats were fed a 20% (w/w) casein control diet, or a diet containing 6%, 14% or 20% chicken protein (CP) for 4 weeks. Choline (**A**), betaine (**B**), dimethylglycine (DMG, **C**), sarcosine (**D**), glycine (**E**), and serine (**F**), were measured in fasting plasma samples. Values shown are means ± standard deviation (*n* = 6). One-way ANOVA with Dunnet’s *post hoc* test was used to determine values significantly different from control (* *p* < 0.05, ** *p* < 0.01, *** *p* < 0.001).

No change in plasma choline was observed in rats fed CP compared to controls (Figure 3A). The plasma concentrations of betaine (Figure 3B), DMG (Figure 3C), sarcosine (Figure 3D), glycine (Figure 3E), and serine (Figure 3F) were increased in the CP-treated rats compared to casein-fed rats.

### 3.2. Hepatic Gene Expression

The hepatic mRNA level of *Mtr* was unchanged, while *Mtrr* showed a small but significant increase in expression. The hepatic gene expression of Cth, the final enzyme in the transsulfuration pathway, was significantly increased 1.4 fold after CP administration. However, the mRNA level of the first enzyme in this pathway, Cbs, was unchanged.

The gene expression of *Pemt* was unchanged. The hepatic mRNA level of the gene encoding *Chka*, *Chkb* and of genes involved in the choline oxidation pathway, *Chdh*, *Bhmt*, *Dmgdh*, *Sardh* and *Gnmt*, were all unchanged in the CP-treated animals. The expression of genes involved in serine and glycine interconversion, *Shmt1* and *Shmt2*, however, was significantly increased (Table 2).

**Table 2:** Hepatic gene expression after 4 weeks of dietary intervention^1^.

Gene		Diet Groups		
Symbol	Name	Control	Chicken Protein	*p*-Value
**Homocysteine Remethylation**				
*Mtr*	Methionine synthase (5-methyltetrahydrofolate-homocysteine methyltransferase)	1.00 ± 0.19	0.98 ± 0.23	0.856
*Mtrr*	Methionine synthase reductase (5-methyltetrahydrofolate-homocystein )	1.00 ± 0.03	1.11 ± 0.07	0.005
**Transsulfuration**				
*Cbs*	Cystathionine-beta-synthase	1.00 ± 0.25	0.94 ± 0.26	0.663
*Cth*	Cystathionase (cystathionine gamma lyase) Cse, Cys3	1.00 ± 0.14	1.41 ± 0.24	0.005
**Choline Synthesis**				
*Pemt*	Phosphatidyletanolamine *N*-methyltransferase	1.00 ± 0.14	1.14 ± 0.12	0.095
*Chka*	Choline kinase (alpha)	1.00 ± 0.21	0.82 ± 0.15	0.119
*Chkb*	Ethanolamine kinase/Choline kinase beta	1.00 ± 0.14	1.17 ± 0.16	0.084
**Choline Oxidation Pathway, Mitochondria**				
*Chdh*	Choline dehydrogenase	1.00 ± 0.10	1.01 ± 0.10	0.899
*Dmgdh*	Dimethylglycine dehydrogenase	1.00 ± 0.29	0.87 ± 0.19	0.377
*Sardh*	Sarcosine dehydrogenase	1.00 ± 0.11	1.18 ± 0.30	0.212
*Shmt2*	Serine hydroxymethyltransferase 2 (mit)	1.00 ± 0.08	1.49 ± 0.25	0.001
**Choline Oxidation Pathway, Cytosol**				
*Bhmt*	betaine-homocysteine *S*-methyltransferase	1.00 ± 0.34	0.76 ± 0.11	0.139
*Gnmt*	Glycine *N*-methyltransferase	1.00 ± 0.21	1.12 ± 0.20	0.338
*Shmt1*	Serine hydroxymethyltransferase (soluble)	1.00 ± 0.14	1.33 ± 0.14	0.002

^1^ Expression levels were normalized to 18 s expression and relative values to control are given as means ± SD (*n* = 6). Results were analyzed by unpaired *t*-test or by Mann Whitney test if not normally distributed according to the Kolmogorov-Smirnov test of normality. *p* < 0.05, significantly different from control values.

### 3.3. Vitamin B Status

**Figure 4:**
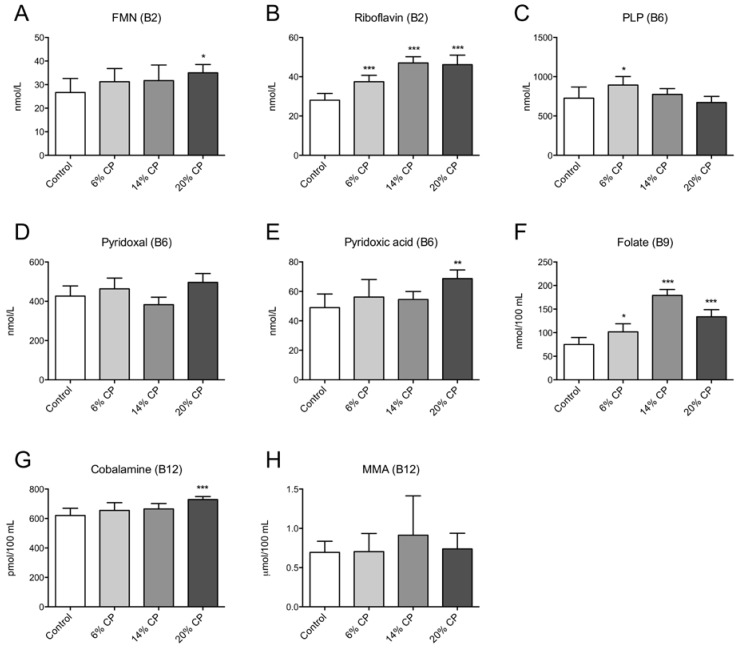
Plasma concentrations of markers of B-vitamin status. Rats were fed a 20% (w/w) casein control diet, or a diet containing 6%, 14% or 20% chicken protein (CP) for 4 weeks. Flavin mononucleotide (FMN, **A**), riboflavin (**B**), pyridoxal phosphate (PLP) (**C**), pyridoxal (**D**), pyridoxic acid (**E**), folate (**F**), cobalamin (**G**), and methylmalonic acid (MMA, **H**), were measured in fasting plasma samples. Values shown are means ± standard deviation (*n* = 6). One-way ANOVA with Dunnet’s *post hoc* test was used to determine values significantly different from control (* *p* < 0.05, ** *p* < 0.01, *** *p* < 0.001).

The plasma concentration of flavin mononucleotide (FMN) and riboflavin was significantly increased in rats fed the 20% CP diet compared to control (Figure 4A,B). The plasma concentration of PLP and pyridoxal was unchanged in CP-fed rats compared to control (Figure 4C,D). However, an increased plasma level of pyridoxic acid was observed in the 20% CP group (Figure 4E). Plasma levels of folate were increased in all CP-fed rats (Figure 4F) while cobalamin was increased only by the 20% CP (Figure 4G). In contrast, the functional marker of vitamin B12, MMA, was unchanged (Figure 4H).

## 4. Discussion

In this study in rats fed a normal fat diet (7%, w/w) we demonstrated that a water-soluble protein extract of chicken (CP) with a low methionine/glycine ratio reduced plasma tHcy and increased levels of serine and metabolites of choline oxidation and transsulfuration compared to a casein diet. The CP diet also affected the status of several B vitamins.

Hcy is produced from methionine during a myriad of methylation reactions, and early experiments demonstrated that curtailed dietary supply of labile methyl groups like methionine and choline, increases the rate of Hcy remethylation back to methionine [26]. Additionally, excess methionine intake has been shown to decrease remethylation of Hcy [27]. Finally, a low methionine-glycine ratio, as in CP compared to casein (0.11 *vs.* 1.57, respectively [11]), has previously been suggested to lower tHcy in rats [1]. Together, this suggests the substantially lower content of methionine in the CP diet to be an important determinant of the lower tHcy observed among the CP fed rats. A probable increase in BHMT activity, a known effect following reduced methionine intake [28], would also help explain the higher concentrations of DMG and sarcosine after CP treatment. Additionally, according to the USDA food composition database (http://ndb.nal.usda.gov), the betaine content of chicken meat from legs and wings is about twice as high as compared to dairy products. This could explain the higher plasma concentration of betaine, and also supports increased BHMT-mediated remethylation of Hcy, leading to increased production of the downstream metabolites DMG and sarcosine. However, the gene expression of BHMT was similar between groups, indicating that a potential altered activity must be related to post-transcriptional regulation.

Although gene expression of MS was unchanged, an increased remethylation by this enzyme cannot be excluded. The CP fed rats showed higher plasma folate compared to control which is associated with lower tHcy levels in humans [29,30], as well as in mouse models [31], generally believed to be mainly related to mTHF being the methyl donor for MS-mediated remethylation of homocysteine. However, reduced Hcy production due to mTHF being an inhibitor of methyltransferases like GNMT [18] seems to be the most important mechanism for this association, as demonstrated in subjects with marginal folate status [32]. We also observed higher levels of vitamin B2, which could increase enzyme activity of important enzymes in the folate cycle, including methylene tetrahydrofolate reductase, responsible for mTHF production, and MS reductase, important for maintaining MS function [33,34]. This would support increased production of mTHF, thus contributing to the Hcy-lowering.

Importantly, the tHcy decrease could also be the consequence of increased Hcy catabolism through the transsulfuration pathway, as the gene expression of Cth was significantly increased. This was accompanied by increased plasma concentrations of cystathionine and cysteine. However, when dietary supply of methionine is low, previous studies have shown that transsulfuration flux decreases [35]. Additionally, CTH mRNA changes were small and may not alter its protein expression.

The concentrations of other metabolites along the choline oxidation pathway, namely DMG, sarcosine, glycine and serine were increased after CP administration. The gene expression of Bhmt, Dmgdh and Sardh was unchanged after CP treatment. Notably, the 20% CP diet contained six-fold higher levels of glycine, while the content of serine was only half compared with the control diet [11]. Thus, we cannot rule out the possibility that the increased level of plasma DMG and sarcosine could be a direct consequence of product inhibition of SARDH and DMGDH, respectively, due to the higher intake of glycine in the CP fed animals. Glycine is also a product of the catabolism of threonine [36], but as the threonine content of CP was much lower than in casein, this was most probably not a significant contributor to the elevated plasma glycine. Serine is used as a substrate for the first enzyme in the transsulfuration pathway, where serine and Hcy condenses to form cystathionine. If transsulfuration flux is reduced to spare Hcy for methionine synthesis, this could contribute to the accumulation of serine. However, an increased expression of Shmt1 and Shmt2 mRNA was observed, which suggest that the higher concentrations of serine observed among the animals receiving the CP diet may also be due to increased conversion from glycine. We observed increased catabolism of vitamin B6, which is used for degradation of glycine through the glycine cleavage system in the mitochondria [37]. Thus, further studies should evaluate if increased B6 catabolism is a mechanism induced to spare glycine for serine production.

## 5. Conclusions

In summary, a water-soluble protein extract of chicken (CP) reduced plasma tHcy and interfered with circulating markers of B vitamin status. The low ratio of methionine to glycine in CP may play an important role due to the importance of regulating methionine availability for methylation reactions, and the higher folate concentrations may have contributed to decreased Hcy synthesis due to its ability to inhibit methyltransferases like GNMT. Further studies are needed to further clarify the specific effects of individual dietary amino acids or different amino acid composition on the metabolic pathways related to Hcy metabolism.
